# Photo-Triggered Reversible Phase Transfer of Azobenzene-Based Ionic Liquid Surfactants between Oil and Water

**DOI:** 10.3390/ijms20071685

**Published:** 2019-04-04

**Authors:** Zhiyong Li, Ying Feng, Xiaoqing Yuan, Huiyong Wang, Yuling Zhao, Jianji Wang

**Affiliations:** Collaborative Innovation Center of Henan Province for Green Manufacturing of Fine Chemicals, Key Laboratory of Green Chemical Media and Reactions, Ministry of Education, School of Chemistry and Chemical Engineering, Henan Normal University, Xinxiang 453007, China; lizhiyong03@126.com (Z.L.); yli@htu.cn (Y.F.); yuanxiaoqingxx@163.com (X.Y.); whyhnxx@163.com (H.W.); ylzhao@htu.cn (Y.Z.)

**Keywords:** ionic liquids, phase transfer, photo-trigger, reversible

## Abstract

The reversible phase transfer of compounds between two immiscible liquid phases has many applications in a wide range of fields, and ionic liquids have been widely used as potential functional solvents and catalysts. However, photo-triggered reversible phase transfer of ionic liquids between the organic phase and water phase has not been reported so far. In the present work, the reversible phase transfer of six kinds of azobenzene-based ionic liquid surfactants between the organic phase and water phase is investigated by alternative irradiation of UV and visible light. Factors affecting the transfer efficiency, such as chemical structure and concentration of the ionic liquid surfactants, equilibrium photo-isomerization degree, and the aggregation state of ionic liquid surfactants are investigated in detail. It is shown that transfer efficiency greater than 89% was achieved under optimal conditions, equilibrium photo-isomerization degree of the ionic liquid surfactants is the main factor to determine their transfer efficiencies, and the aggregation of *cis*-isomers is not beneficial for the transfer.

## 1. Introduction

Phase-transfer processes of molecules, supramolecular assemblies, and particles between two immiscible liquid phases are of critical importance to many applications, and have attracted much attention in a variety of fields such as catalyst recycling [1,2], nanoparticles synthesis and application [3,4,5], and transport across biological membranes [6,7]. Among these investigations, reversible phase transfer, especially environmental stimulus responsive reversible phase transfer is a very important research field. For example, by alternative bubbling and removal of CO_2_ [8,9,10,11], the catalyst could move back and forth between water and organic phases, simplifying the process for recycling the catalyst. Phase transfer driven by temperature is another important strategy where nanoparticles [12,13], functionalized ionic liquids [14], and nanogels [15] can reversibly transfer between an organic phase and an aqueous phase in response to temperature changes. Moreover, by adjusting the pH value of the solution, the compounds with acid or base functional groups [13,16,17] may reversibly transfer between the organic phase and water phase.

The use of light as an external stimulus for stimuli-responsive phase transfer is of particular interest because light has a noninvasive character and can be delivered remotely and precisely in space and time [18,19,20]. Recently, some valuable work has been reported on the light-responsive phase transfer. It is shown that a homogeneous ruthenium-carbene catalyst can be recycled by a light-controlled reversible transition of a nitrobenzospiropyran unit [1]. Yang et al. [2] reported a photo-responsive polyoxometalate complex catalyst, which might be recycled through a simple photo-driven phase transfer. The composite colloids grafted by a photo-responsive spiropyran group [21] could have a reversible transfer between toluene and water. Peng et al. [22] performed a reversible phase transfer of α-cyclodextrin coated gold nano-particles between water and toluene phases, which could be modulated by UV and visible light. These studies bring new insights into the research of environmental stimulus responsive phase transfer processes.

In the past two decades, ionic liquids (ILs) have been developed with great interest because of their remarkable physical properties like negligible volatility, low melting point, high stability, and strong capacity to dissolve many materials [23]. Another attractive feature of ILs is the designable ability of their structures and properties [24,25]. Using this unique feature, many new ILs with improved properties can be created. Thus, it should be interesting to produce a class of photo-responsive ILs by introducing photo-responsive groups. As one of the common light-responsive groups, azobenzene derivatives have high medium sensitive and reversible isomerization performance upon UV/vis irradiation [26,27,28]. The *trans*-isomer of azobenzene groups has a linear structure, and its polarity is small due to the axial symmetry, while the bent structure of *cis*-isomer leads to an increased dipolar moment because of its non-axial symmetry [20]. Upon UV light irradiation, *trans*-azobenzene is converted to *cis*-azobenzene [19], this configuration change provides a favorable feature for controlling hydrophilicity/hydrophobicity of the azobenzene-based compounds. Therefore, it is possible to design photo-responsive ILs containing an azobenzene unit for reversible transfer between the organic phase and water phase. However, to the best of our knowledge, photo-triggered reversible phase transfer of ILs between the organic phase and water is not reported so far.

**Figure 1:**
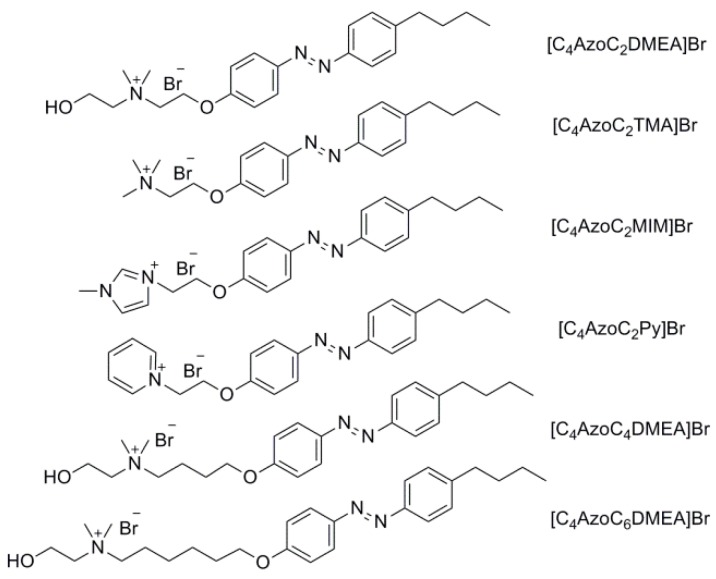
Chemical structures of the azobenzene-based ILs surfactants.

In this work, six kinds of such light-responsive IL surfactants have been synthesized and their chemical structure and abbreviation of name are shown in Figure 1. The reversible phase transfer between the organic phase and water phase were investigated systematically. The chemical structure and concentration of them, the equilibrium photo-isomerization efficiency and the photo-regulated aggregation of the IL surfactants were used to discuss the photo-triggered reversible phase transfer of them.

## 2. Results and Discussion

### 2.1. Photo-Triggered Reversible Phase Transfer of the Azobenzene-Based IL Surfactants

**Figure 2:**
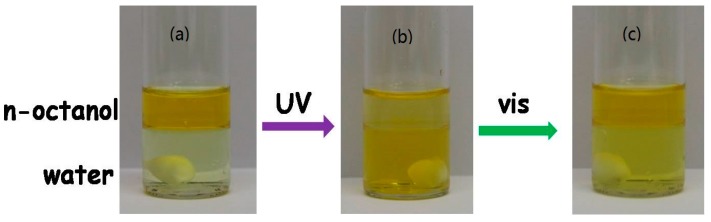
Photos of the reversible phase transfer of [C_4_AzoC_2_DMEA]Br between n-octanol and water by UV/vis irradiation, the concentration of [C_4_AzoC_2_DMEA]Br is 1.0 × 10^−2^ mol/kg: (**a**) before UV irradiation; (**b**) after UV irradiation; (**c**) after further vis irradiation.

As an example, the photo-induced phase transfer of [C_4_AzoC_2_DMEA]Br between n-octanol and water was investigated. It was found that when the two immiscible solvents were mixed (at the phase volume ratio of organic phase to water of 1:2) with [C_4_AzoC_2_DMEA]Br in one glass vessel at 25.0 °C, a photo-driven phase transfer of the azobenzene-based IL surfactant was observed (Figure 2). At the initial stage, a yellow n-octanol phase was formed at the top of the vessel, while a colorless water phase was formed at the bottom (Figure 2a). This indicates that at that moment, [C_4_AzoC_2_DMEA]Br was mainly located in the organic phase. However, after the whole system was irradiated with UV light for 30 min at 25.0 °C under stirring, the bottom water phase became colored, but the color of the top n-octanol layer was getting lighter (as shown in Figure 2b, the molar absorption coefficient of the *cis*-isomer of the azobenzene compound is larger than that of the *trans*-isomer causing organic phase which shows color in Figure 2b), which means that a portion of [C_4_AzoC_2_DMEA]Br was transferred from n-octanol to water. As confirmed by UV-vis spectral measurements (as shown in Figure S7), the transfer efficiency of [C_4_AzoC_2_DMEA]Br was greater than 70%. Interestingly, this part of [C_4_AzoC_2_DMEA]Br was returned to n-octanol phase from water after further irradiation by visible light (Figure 2c).

From the view of the chemical structure of [C_4_AzoC_2_DMEA]Br, azobenzene is a hydrophobic group and can be strongly solvated by n-octanol, which is not beneficial for the transfer of [C_4_AzoC_2_DMEA]Br from n-octanol to the water phase. In order to obtain a higher transfer efficiency of [C_4_AzoC_2_DMEA]Br, the strategy by adding co-solvent into n-octanol was used in the next experiments. For this purpose, a poor solvent for [C_4_AzoC_2_DMEA]Br, n-hexane was selected and added into n-octanol to regulate solubility of the IL surfactant in the organic phase. It can be seen from Table 1 that the transfer efficiency of [C_4_AzoC_2_DMEA]Br increased notably from 70.8 to 86.6% by using mixed organic solvent phase (n-octanol/n-hexane = 2:1, *v*/*v*) at the same phase volume ratio of organic phase to water. Moreover, the transfer efficiency of [C_4_AzoC_2_DMEA]Br increased with decreasing phase volume ratio of organic phase to water from 1:1 to 1:2.5, indicating that the increase of water phase volume is beneficial for the transfer of the IL surfactant from the organic phase to water phase. Then, the water content in the organic phase (n-octanol/n-hexane = 2:1, *v*/*v*) before and after UV irradiation was determined by a Karl Fisher method to evaluate the composition changes of the organic phase. It was shown that the water content in the organic phase before and after UV irradiation was 0.83% and 1.54% (*w*/*w*), respectively. This result indicates that upon light irradiation, only azobenzene-based IL surfactants shuttled between the organic phase and water phase, there was no change in the composition of the solvents. In addition, the azobenzene-based IL surfactants could also transfer between organic phase (n-octanol + toluene, n-octanol + cyclohexane, or n-octanol + ethyl acetate) and water. However, the solubility of them in these organic solvents was low, and the transfer efficiency was not high (not shown).

**Figure 3:**
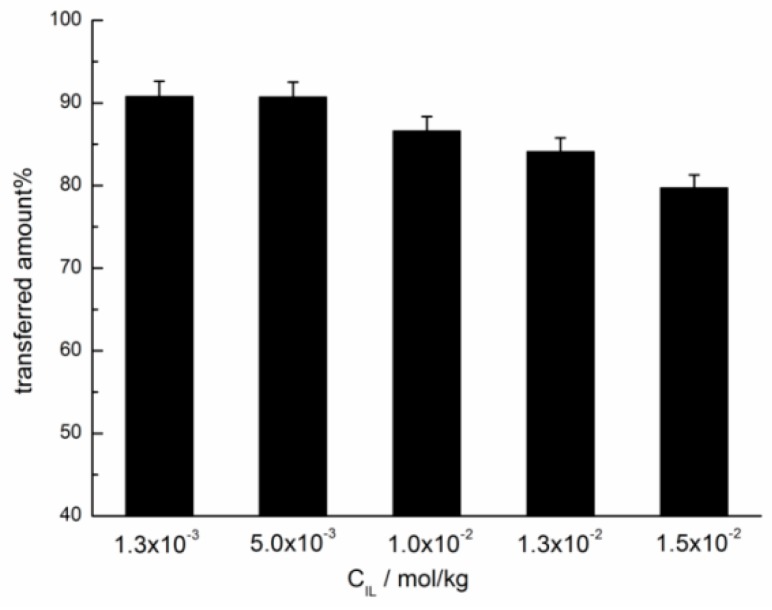
The transfer efficiency of [C_4_AzoC_2_DMEA]Br at different concentrations after UV irradiation at 25.0 °C.

The effect of concentration of [C_4_AzoC_2_DMEA]Br on its transfer efficiency was investigated in detail, and the results were shown in Figure 3. It is shown that in the investigated concentration range (from 1.3 × 10^−3^ to 1.5 × 10^−2^ mol/kg), the transfer efficiency of [C_4_AzoC_2_DMEA]Br increased with the decrease of the concentration. This trend means that a high concentration of the IL surfactant is not beneficial for the transfer from the organic phase to water phase. Moreover, the solubility of the azobenzene-based IL surfactants in water was determined by a spectrophotometric method at 25.0 °C, and the results were shown in Table S1 in supporting information. It was shown that solubility of these ionic liquid surfactants was much higher than the concentration of the ionic liquid surfactants transferred from the organic phase to water phase. Therefore, the transfer of azobenzene-based IL surfactants from the organic phase to water was not controlled by the solubility of them.

**Figure 4:**
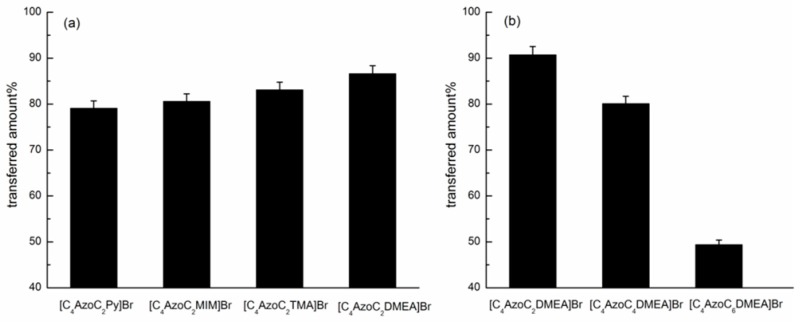
Effect of chemical structure of the azobenzene-based IL surfactants on the transfer efficiency from the organic phase to water phase after UV irradiation at 25.0 °C: (**a**) 1.0 × 10^−2^ mol/kg; (**b**) 5.0 × 10^−3^ mol/kg.

Furthermore, the chemical structure of the azobenzene-based IL surfactants on the transfer efficiency was also investigated, and the results were shown in Figure 4. It can be seen that the transfer efficiency of the IL surfactants decreased in the order: [C_4_AzoC_2_DMEA]Br > [C_4_AzoC_2_TMA]Br > [C_4_AzoC_2_MIM]Br > [C_4_AzoC_2_Py]Br; and [C_4_AzoC_2_DMEA]Br > [C_4_AzoC_4_DMEA]Br > [C_4_AzoC_6_DMEA]Br. From the viewpoint of chemical structure, [C_4_AzoC_2_DMEA]Br, [C_4_AzoC_2_TMA]Br, [C_4_AzoC_2_Py]Br and [C_4_AzoC_2_MIM]Br have different head groups of the cations, while [C_4_AzoC_2_DMEA]Br, [C_4_AzoC_4_DMEA]Br and [C_4_AzoC_6_DMEA]Br have different alkyl spacer lengths between the azobenzene group and head group. Therefore, by using the above transfer efficiency data, the effect of alkyl spacer length and head group type on the transfer efficiency could be examined. The IL surfactant has a relatively hydrophobic head group (1-methylimidazole or pyridine) or a longer alkyl spacer length of the cation is not benefit for its transfer between the organic phase and water phase.

As a selected example, the reversible transfer process of [C_4_AzoC_2_DMEA]Br between the organic phase and the water phase was investigated by alternative irradiation of UV and visible light, and the results were shown in Figure S8. The phase transfer could be recycled at least five times without significantly altering the absorbance in organic solvent and water. Therefore, a reversible phase transfer was confirmed.

### 2.2. Photo-Isomerization of the Azobenzene-Based IL Surfactants

**Figure 5:**
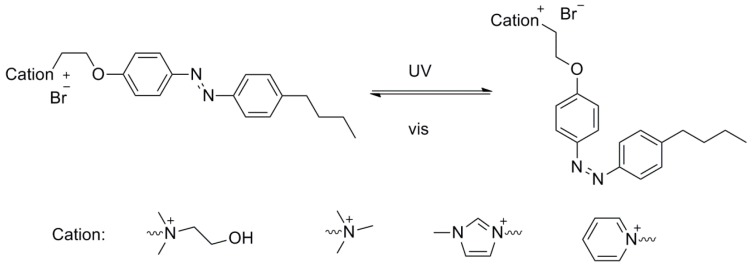
Light-induced isomerization of azobenzene-based IL surfactants.

**Figure 6:**
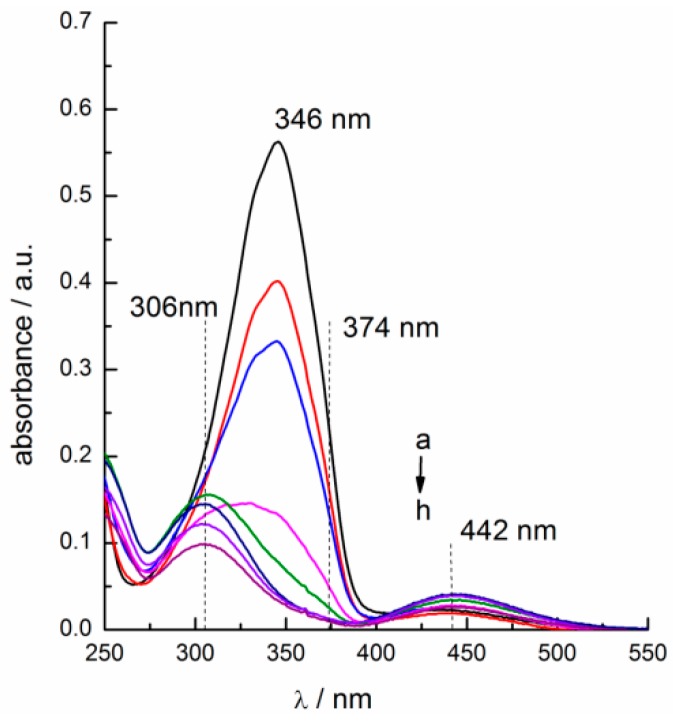
UV-vis spectra of 1.0 × 10^−2^ mol/kg [C_4_AzoC_2_DMEA]Br in n-octanol + n-hexane (2:1, *v*/*v*) with different UV irradiation time at 25.0 °C: a, initial state; b, 2 min; c, 4 min; d, 8 min; e, 10 min; f, 20 min; g, 30 min; h, 60 min.

As discussed above, the transfer of azobenzene-based IL surfactants can be regulated by alternative irradiation of UV and vis-light. The possible reason is that isomerism of the azobenzene groups can regulate the hydrophilicity of the photo-responsive compounds [21,29], which affects their transfer between the organic phase and water phase. For the compounds investigated in the present work, as shown in Figure 5, the isomerization from *trans*- to *cis*-isomer under UV irradiation led to the increase of hydrophilicity of the IL surfactant, thus facilitating the transfer of them from the organic phase to the water phase. On the other hand, by alternatively irradiating with visible light, the azobenzene-based IL surfactants could move back to organic phase because of the reverse isomerization of azobenzene group from *cis-* to *trans*-isomer. Therefore, the transfer of the photo-responsive IL surfactants between the organic phase and water phase originates from the increase in hydrophilicity of them after isomerism of its azobenzene groups [30]. The degree of isomerization of the IL surfactants from *trans-* to *cis*-isomers would have an important effect on the transfer efficiency of them. To have a deep understanding of the photo-isomerism on the phase transfer, factors affecting the photo-isomerism behavior of the IL surfactants were investigated by UV-vis spectroscopy. For instance, Figure 6 showed the UV-vis spectrum of [C_4_AzoC_2_DMEA]Br in n-octanol + n-hexane (2:1, *v*/*v*) at a different UV irradiation time. The UV-vis spectra for [C_4_AzoC_2_MIM]Br, [C_4_AzoC_2_TMA]Br, [C_4_AzoC_2_Py]Br, [C_4_AzoC_4_DMEA]Br, and [C_4_AzoC_6_DMEA]Br in the same mixed organic phase were presented in Figures S9–S14 (Supplementary Materials).

As shown in Figure 6, the maximum absorption peak at 346 nm originates from the π→π* transition of *trans*-isomer. Upon continuous UV light irradiation, the absorbance intensity at 346 nm gradually decreased and finally disappeared, indicating the disappearance of *trans*-isomer in the system. Meanwhile, two new absorption peaks came out at 306 and 442 nm, which were resulted from the π→π* and n→π* transition of *cis*-isomer, respectively [19]. Similar experimental results were also obtained from Figures S9–S14 in the supporting information. After UV irradiation, minimal absorption of *cis*-form at 374 nm was precisely between the peaks at 306 and 442 nm. Zakrevskyy et al. [31] assumed that the initial state is pure *trans*-isomer [18,31] before irradiation and *cis*-isomer at 374 nm is negligible; thus, the absorbance at 374 nm could be used to calculate the content of *trans*-isomers after the isomerization. Thus, the equilibrium isomerization efficiencies of them were listed in Table 2.

It can be seen from Table 2 that the chemical structure of the IL surfactants could affect the equilibrium isomerization efficiencies of them in the organic phase. For the IL surfactants with the same alkyl spacer length, the equilibrium isomerization efficiencies decreased in the order: [C_4_AzoC_2_DMEA]Br > [C_4_AzoC_2_TMA]Br > [C_4_AzoC_2_MIM]Br > [C_4_AzoC_2_Py]Br, while for the IL surfactants with the same head group of the cation, but different alkyl spacer length, the equilibrium isomerization efficiencies decreased in the order: [C_4_AzoC_2_DMEA]Br > [C_4_AzoC_4_DMEA]Br > [C_4_AzoC_6_DMEA]Br. Compared with the results reported in Figure 4, the trends in the isomerization efficiency of them were in agreement with that in the transfer efficiency reported above. These results showed that the photo-induced equilibrium isomerization efficiency of the IL surfactants played a significant role in the transfer efficiency between the organic phase and the aqueous phase.

However, it should be noted that for [C_4_AzoC_2_DMEA]Br, the equilibrium isomerization efficiencies at 1.0 × 10^−2^ mol/kg and 5.0 × 10^−3^ mol/kg were 95.2% and 94.9%, respectively. These results indicated that the difference of equilibrium isomerization efficiencies of the IL surfactants was small at different concentrations, which is not in accordance with their transfer efficiencies (the difference of them is greater than 4%) shown in Figure 3. Moreover, the equilibrium isomerization efficiencies of [C_4_AzoC_2_DMEA]Br, [C_4_AzoC_4_DMEA]Br and [C_4_AzoC_6_DMEA]Br at 5.0 × 10^−3^ mol/kg were 94.9, 93.1, and 91.5%, respectively. This indicates that alkyl spacer length between the head group and azobenzene unit did not have a significant effect on the equilibrium isomerization efficiencies. However, as shown in Figure 4b, the transfer efficiencies of them were 90.7, 80.1, and 49.4%, respectively, at the same concentration. These results indicate that the photo-isomerization degree of the IL surfactants is the main factor, but it is not the only factor to affect the transfer efficiencies of them.

### 2.3. Photo-Regulated Aggregation of the IL Surfactants

**Figure 7:**
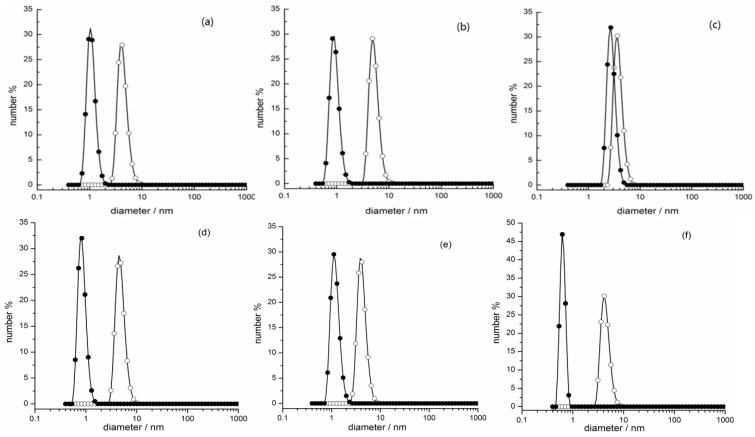
DLS results of 5.00 × 10^−3^ mol/kg azobenzene-based IL surfactants in aqueous solution at 25.0 °C: (**a**) [C_4_AzoC_2_DMEA]Br; (**b**) [C_4_AzoC_4_DMEA]Br; (**c**) [C_4_AzoC_6_DMEA]Br; (**d**) [C_4_AzoC_2_TMA]Br; (**e**) [C_4_AzoC_2_MIM]Br; (**f**) [C_4_AzoC_2_Py]Br; ○, before UV irradiation; ●, after UV irradiation.

The above section clearly shows that the photo-induced phase transfer of azobenzene-based IL surfactants cannot be interpreted only from their equilibrium photo-isomerization efficiencies. From solution chemistry, solvation and aggregation of the compounds in different solvents may have an important effect on their transfer between the immiscible two phases. To have a deeper understanding of the phase transfer of the azobenzene-based IL surfactants, the aggregation of the *trans*-isomer and *cis*-isomer of [C_4_AzoC_2_TMA]Br, [C_4_AzoC_2_MIM]Br, [C_4_AzoC_2_Py]Br, [C_4_AzoC_2_DMEA]Br, [C_4_AzoC_4_DMEA]Br, and [C_4_AzoC_6_DMEA]Br in n-octanol + n-hexane (2:1, *v*/*v*) and water before and after UV light irradiation was investigated by dynamic light scattering (DLS) and conductivity titrations at 25.0°C, and the results were shown in Figure 7 and Figure S15. It can be seen from Figure S15 that the size distribution was around 1 nm, indicating that these IL surfactants cannot form aggregates in the mixed organic phase before UV irradiation [32]. Furthermore, no obvious change in the size distribution was observed for these IL surfactants after UV irradiation. This result indicates that UV irradiation only has a small impact on the aggregation states of *trans*-isomer and *cis*-isomer of azobenzene-based IL surfactants in the organic phase.

**Figure 8:**
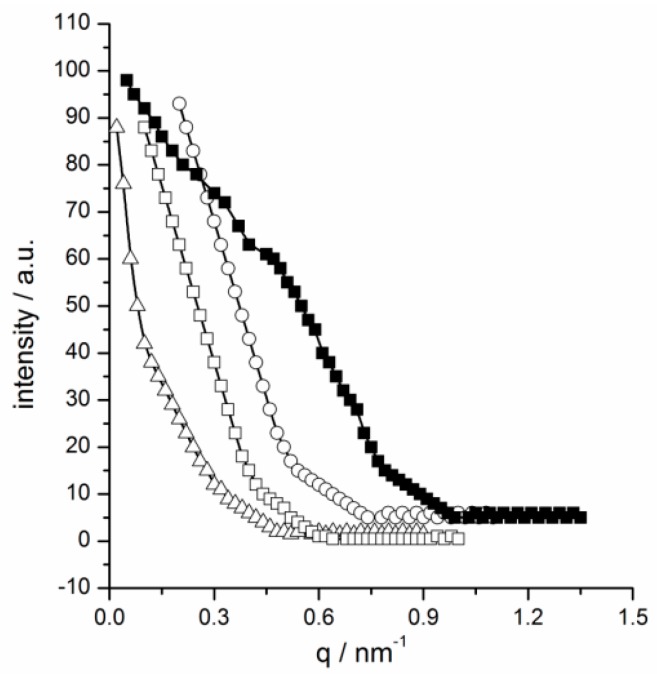
SAXS data for 5.0 × 10^−3^ mol/kg IL surfactants in aqueous solution at 25.0 °C: ○, [C_4_AzoC_2_DMEA]Br before UV irradiation; △, [C_4_AzoC_4_DMEA]Br before UV irradiation; □, [C_4_AzoC_6_DMEA]Br before UV irradiation; ■, [C_4_AzoC_6_DMEA]Br after UV irradiation.

Nevertheless, it is clear from Figure 7 that the size of [C_4_AzoC_2_TMA]Br, [C_4_AzoC_2_MIM]Br, [C_4_AzoC_2_Py]Br, [C_4_AzoC_2_DMEA]Br, [C_4_AzoC_4_DMEA]Br, and [C_4_AzoC_6_DMEA]Br in aqueous solution was about 5 nm at 25.0°C before UV irradiation, which can be attributed to the formation of aggregates of *trans*-isomer in water [2]. Interestingly, the aggregates of [C_4_AzoC_2_TMA]Br, [C_4_AzoC_2_MIM]Br, [C_4_AzoC_2_Py]Br, [C_4_AzoC_2_DMEA]Br, and [C_4_AzoC_4_DMEA]Br were destroyed after UV irradiation (see conductivity curves in Figures S16–S19 in Supplementary Materials), while the aggregates of *cis*-isomer of [C_4_AzoC_6_DMEA]Br were maintained in aqueous solution under the same conditions. Furthermore, as the selected examples, small-angle X-ray scattering (SAXS) was used to investigate the change in the aggregation behavior of [C_4_AzoC_2_DMEA]Br, [C_4_AzoC_4_DMEA]Br, and [C_4_AzoC_6_DMEA]Br in water and the mixed organic solvents, and the results were shown in Figure 8. It was found that, the SAXS data of [C_4_AzoC_2_DMEA]Br, [C_4_AzoC_4_DMEA]Br, and [C_4_AzoC_6_DMEA]Br in the organic mixtures could not be fitted by the micelle model, indicating that [C_4_AzoC_2_DMEA]Br, [C_4_AzoC_4_DMEA]Br, and [C_4_AzoC_6_DMEA]Br could not form aggregates in the mixed organic phase. For [C_4_AzoC_2_DMEA]Br, [C_4_AzoC_4_DMEA]Br, and [C_4_AzoC_6_DMEA]Br in water, SAXS results showed that they formed spherical micelle before UV irradiation, and the size of the aggregates of [C_4_AzoC_2_DMEA]Br, [C_4_AzoC_4_DMEA]Br, and [C_4_AzoC_6_DMEA]Br was 4, 6, and 5 nm, respectively. Interestingly, the aggregate of [C_4_AzoC_2_DMEA]Br and [C_4_AzoC_4_DMEA]Br was destroyed upon UV irradiation, while the size of the aggregate of *cis*-[C_4_AzoC_6_DMEA]Br was changed to 3 nm. These results are consistent with that obtained by DLS. The formation of aggregates of *cis*-[C_4_AzoC_6_DMEA]Br in aqueous solution after UV irradiation means that water has weak solvation ability for *cis*-[C_4_AzoC_6_DMEA]Br, which is not beneficial for the transfer from the organic phase to aqueous phase. This is why [C_4_AzoC_2_DMEA]Br and [C_4_AzoC_4_DMEA]Br have a much higher transfer efficiency than [C_4_AzoC_6_DMEA]Br.

In addition, the aggregation results could be used to explain the effect of IL surfactant concentration on transfer efficiency of [C_4_AzoC_2_DMEA]Br presented in Figure 3. Our previous work [30] shows that the critical aggregation concentration (CAC) of [C_4_AzoC_2_DMEA]Br in water is 2.30 × 10^−3^ mol/kg before UV irradiation, and the CAC value increases to 1.30 × 10^−2^ mol/kg after UV irradiation (see Figure S16 in Supplementary Materials). This result suggests that *trans*-[C_4_AzoC_2_DMEA]Br could form aggregates in water before UV irradiation within the concentration range investigated here. Upon UV irradiation, the aggregates of [C_4_AzoC_2_DMEA]Br at 5.00 × 10^−3^, 1.00 × 10^−2^ and 1.30 × 10^−2^ mol/kg were destroyed, while *trans*-[C_4_AzoC_2_DMEA]Br could form aggregates at 1.5 × 10^−2^ mol/kg after UV irradiation. The formation of aggregates of *cis*-isomer was not beneficial to its transfer from the organic phase to the water phase. This is why [C_4_AzoC_2_DMEA]Br has a lower transferred efficiency at the higher concentration.

From the chemical structure of the azobenzene-based compounds, UV irradiation may enhance their polarity, and then induce the changes in the solvation state of these compounds. To evaluate the effect of the changes in the polarity on the transfer of azobenzene-based IL surfactants, the polarity of [C_4_AzoC_2_DMEA]Br, [C_4_AzoC_4_DMEA]Br, and [C_4_AzoC_6_DMEA]Br in n-octanol + n-hexane (2:1, *v*/*v*) and water before and after UV light irradiation was determined by Reichardt’s betaine dye method [33]. The calculated *E*_T_(30) values were listed in Table 3 and empirically used to evaluate the polarity of the IL surfactants in solution. It is shown that the *E*_T_(30) values of the IL surfactants in solution increased after UV irradiation, and the increment of *E*_T_(30) values decreased in the order [C_4_AzoC_2_DMEA]Br > [C_4_AzoC_4_DMEA]Br > [C_4_AzoC_6_DMEA]Br, which means that polarity of the IL surfactants decreased with increasing alkyl spacer of the azobenzene-based IL surfactants. This result could be used to explain the effect of alkyl spacer length of the IL surfactants on their transfer efficiencies presented in Figure 4b, azobenzene-based IL surfactant with a shorter alkyl spacer has a higher transfer efficiency.

## 3. Materials and Methods

### 3.1. Chemicals

*N*,*N*-dimethyl-ethanolamine (99%), 1-methyl-imidazole (99%), 1,4,7,10,13,16-hexaoxacyclooctadecane (99%), phenol (99.9%), n-octanol (99%), n-hexane (99%), methanol (99%), *N*,*N*-dimethylformamide (DMF, 99%), dimethyl sulfoxide (DMSO, 99%) were purchased from Shanghai Aladdin and used without further purification. Petroleum ether (b.p. 60–90 °C), 1,2-dibromoethane (99%), 1,6-dibromohexane (99%), 1,4-dibromobutane (99%), acetonitrile (99.9%), pyridine (99%), 4-butylaniline (98%), trimethylamine (30% in ethanol), sodium nitrite (99%), hydrochloric acid (36%), ethyl acetate (99.9%), and sodium carbonate (99%) were commercial products from Shanghai Macklin Company.

The azobenzene-based IL surfactants were prepared and purified by using the procedures described in our previous work [30]. The chemical structures of them were confirmed by ^1^H NMR, and their purity was found to be greater than 97% in mass fraction. The ^1^H NMR spectrum and detailed spectral data are shown in Figures S1–S6 in Supplementary Materials. The melting point of them have been determined by a Netzsch 204F1 DSC, and the results were shown in Table S2 in the Supplementary Information.

### 3.2. UV-vis Spectrum Measurements

UV-vis spectrum of the IL surfactants in n-octanol at different UV irradiation states was investigated by using a UV-4100 spectrophotometer at room temperature. The spectrum was scanned in a wavelength range from 250 to 550 nm, and the solvent was used as the blank. A 365 nm UV light with intensity 100 mW/cm^2^ was used to irradiate the samples at 25.0 ± 0.1 °C under stirring. After photo-isomerization, in order to prevent the transformation from *cis*- to *trans*-isomer, all of the solutions were wrapped with aluminum foil. Reversibility of the isomerism process of the ILs was studied by irradiation with an 18 W LED lamp as visible light.

### 3.3. Partitioning of the Azobenzene-Based IL Surfactants in the Biphasic Oil/Water Systems

As a selected example, [C_4_AzoC_2_DMEA]Br was triggered by UV/vis light irradiation to shuttle back and forth between n-octanol and water. First, a given volume of n-octanol solution containing 5.0 × 10^−^^3^ mol/kg of [C_4_AzoC_2_DMEA]Br was added into water. Then, a cold LED UV light source was used to irradiate the mixture for 30 min at 25.0 ± 0.1°C under stirring. A fraction of the solution was taken out separately from water and n-octanol phases for UV analysis to obtain the concentration of [C_4_AzoC_2_DMEA]Br in n-octanol and water phases. Another batch of the sample was further irradiated by vis-light for 1 h at 25.0 ± 0.1°C under stirring after the UV irradiation. The concentrations of [C_4_AzoC_2_DMEA]Br in n-octanol and water phases were determined again.

The concentrations of [C_4_AzoC_2_DMEA]Br in both phases were determined by measuring the absorbance at 346 nm using a UV-4100 spectrophotometer. The transfer efficiency of [C_4_AzoC_2_DMEA]Br was calculated by Equation (1):

E% = (1 − C_oil_V_oil_/C_0_V_0_) × 100% (1)

where V_oil_ and C_oil_ are the volumes of the organic phase and concentration of [C_4_AzoC_2_DMEA]Br in the organic phase after UV irradiation, and V_0_ and C_0_ are the volume of the organic phase and concentration of [C_4_AzoC_2_DMEA]Br in organic phase before UV irradiation, respectively. Partitioning of the other IL surfactants in biphasic n-octanol/water systems was similarly performed. In the proceeded investigations, n-octanol and n-hexane, instead of n-octanol, were used, and the other conditions remained unchanged.

### 3.4. Dynamic Light Scattering Measurements

The size of the aggregates were determined at 25.0 °C by dynamic light scattering (DLS) technique using a Malvern Zetasizer Nano-ZS 90 light scattering instrument. The scattering angle was 90°. Light of λ = 633 nm from a solid-state He-Ne laser (4.0 mW) was used as the incident beam. In order to avoid the influence of possible dust in the solvent on the experimental results, all samples were filtered by a 0.45 μm filter and placed overnight before the measurement. The refractive index of the mixed solvent used in the experiment was 1.41452, and the viscosity of the solvent was 2.083 mPa·s.

### 3.5. Small Angle X-ray Scattering Measurements

Small-angle X-ray scattering experiments were performed at 25.0 °C by an Anton Paar SAXS Space scattering instrument equipped with a Kratky block-collimation system. The samples were placed in a quartz capillary. The X-ray was generated using a generator with Cu target and the wavelength was 0.1542 nm.

### 3.6. Polarity Measurements of Organic Phase and Water Phase Containing Azobenzene-Based IL Surfactants

*E*_T_(30) values of the systems were empirically used to evaluate the polarity of the organic phase and water phase before and after UV irradiation by the Reichardt’s calculation. The absorption of Reichardt’s dye (33) was used as the probe dye to calculate *E*_T_(30) value of the system by the following Equations [33]:

*E*_T_(33)/kcal·mol^−1^ = 28,591/(*λ*_max_/nm) (2)

*E*_T_(30) = 0.9953*·E*_T_(33) − 8.1132 (3)

In the experiments, Reichardt’s dye (33) was dissolved in CH_2_Cl_2_, and 0.05 mL of the probe dye solution was transferred into a glass vessel. After CH_2_Cl_2_ was evaporated entirely by high purity of argon gas, 3.20 mL of sample was added into the glass vessel under stirring. The solvatochromic experiments were carried out by a UV-4100 spectrophotometer.

## 4. Conclusions

In summary, the phase transfer of six kinds of azobenzene-based IL surfactants between the organic phase and water phase was investigated. It was found that the photo-responsive IL surfactants could be transferred from the organic phase to the aqueous phase by UV irradiation, and they would move back to the organic phase from water by subsequent irradiation of visible light. Using alternative irradiation of UV and visible light, IL surfactants exhibited reversible phase transfer between the organic phase and the aqueous phase. The transfer efficiency of the IL surfactants decreased in the order: [C_4_AzoC_2_DMEA]Br > [C_4_AzoC_2_TMA]Br > [C_4_AzoC_2_MIM]Br > [C_4_AzoC_2_Py]Br, and [C_4_AzoC_2_DMEA]Br > [C_4_AzoC_4_DMEA]Br > [C_4_AzoC_6_DMEA]Br. The IL surfactant has a relatively hydrophobic head group or a longer alkyl spacer length in the cation and is not beneficial for its transfer between the organic phase and water phase.

The equilibrium photo-isomerization efficiency of the IL surfactants is the main factor to affect their transfer efficiencies. For the IL surfactants with the same alkyl spacer length, their transfer efficiency is in agreement with their equilibrium isomerization efficiencies, while for the IL surfactants with the same head group of the cation but different alkyl spacer length, the transfer efficiency of them decreased with the increase of the alkyl spacer lengths. Moreover, the aggregation of *cis*-isomers in water is not beneficial for the transfer of IL surfactants from the organic phase to the water phase. Therefore, in order to obtain a high transfer efficiency, it is necessary to control the concentration of IL surfactant below its *CAC* value of *cis*-isomer in water. These systems are expected to have potential for the recovery of catalysts from products and solvents.

## Figures and Tables

**Table 1:** The transfer efficiency of [C_4_AzoC_2_DMEA]Br from the organic phase to water after UV irradiation at 25.0 °C.

Organic Phase	R	IL Transfer Efficiency %
n-octanol	1:2	70.8
Mixed organic solvent ^1^	1:1	66.0
Mixed organic solvent ^1^	1:1.5	81.1
Mixed organic solvent ^1^	1:2	86.6
Mixed organic solvent ^1^	1:2.5	89.5

^1^ Mixed organic solvent is n-octanol + n-hexane = 2:1 (*v*/*v*); R is the phase volume ratio of organic phase to water; concentration of [C_4_AzoC_2_DMEA]Br in the organic phase is 1.0 × 10^−2^ mol/kg; the uncertainty of the transfer efficiency is within 2%.

**Table 2:** The equilibrium isomerization efficiency of the azobenzene-based IL surfactants in n-octanol + n-hexane (2:1, *v*/*v*) after UV irradiation at 25.0 °C.

Compound	Isomerization Efficiency %
[C_4_AzoC_2_Py]Br ^1^	92.9
[C_4_AzoC_2_MIM]Br ^1^	93.6
[C_4_AzoC_2_TMA]Br ^1^	94.2
[C_4_AzoC_2_DMEA]Br ^1^	95.2
[C_4_AzoC_2_DMEA]Br ^2^	94.9
[C_4_AzoC_4_DMEA]Br ^2^	93.1
[C_4_AzoC_6_DMEA]Br ^2^	91.5

^1^ the concentration of the IL surfactants in the organic phase is 1.0 × 10^−2^ mol/kg; ^2^ the concentration of the IL surfactants in the organic phase is 5.0 × 10^−3^ mol/kg, the isomerization equilibrium time is about 30 min, the uncertainty of the transfer efficiency is within 1%.

**Table 3:** *E*_T_(30) values (in kcal/mol) of IL surfactants in the organic phase and water phase before and after UV irradiation at 25.0 °C.

Compound	Organic Phase			Water Phase		
	Before UV Irradiation	After UV Irradiation	δ*E*_T_(30)	Before UV Irradiation	After UV Irradiation	δ*E*_T_(30)
[C_4_AzoC_2_DMEA]Br	82.3	83.8	1.5	83.2	84.7	1.5
[C_4_AzoC_4_DMEA]Br	82.3	83.5	1.2	82.9	84.3	1.4
[C_4_AzoC_6_DMEA]Br	82.9	83.5	0.6	83.5	84.3	0.8

To avoid interference of IL surfactants on Reichardt’s dye absorption peaks, the concentration of IL surfactants in solution is 2.2 × 10^−5^ mol/kg.

## Supplementary Materials

Supplementary materials can be found at https://www.mdpi.com/1422-0067/20/7/1685/s1.

## Abbreviations

[C_4_AzoC_2_DMEA]Br	2-(4-((4-butylphenyl)diazenyl)phenoxy)-*N*-(2-hydroxyethyl)-*N*,*N*-dimethyl-ethanaminium bromide
[C_4_AzoC_4_DMEA]Br	4-(4-((4-butylphenyl)diazenyl)phenoxy)-*N*-(2-hydroxyethyl)-*N*,*N*-dimethylbutanaminium bromide
[C_4_AzoC_6_DMEA]Br	6-(4-((4-butylphenyl)diazenyl)-phenoxy)-*N*-(2-hydroxyethyl)-*N*,*N*-dimethylhexanaminium bromide
[C_4_AzoC_2_TMA]Br	2-(4-((4-butylphenyl)diazenyl)phenoxy)-*N*,*N*,*N*-trimethylethanaminium bromide
[C_4_AzoC_2_MIM]Br	3-(2-(4-((4-butylphenyl)diazenyl)phenoxy)ethyl)-1-methyl-imidazolium bromide
[C_4_AzoC_2_Py]Br	1-(2-(4-((4-butylphenyl)diazenyl) phenoxy)ethyl)pyridinium bromide
