# Supplementary material for: Photo-Triggered Reversible Phase Transfer of Azobenzene-Based Ionic Liquid Surfactants between Oil and Water
Source: Int J Mol Sci. 2019 Apr 4;20(7):1685. doi: 10.3390/ijms20071685 (PMC6479859; doi:10.3390/ijms20071685)
Supplement: Supplementary file 1 [file ijms-20-01685-s001.pdf]

International Journal of *Molecular Sciences*

Photo-triggered Reversible Phase Transfer of Azobenzene-based Ionic Liquids between Oil and Water

Zhiyong Li, Ying Feng, Xiaoqing Yuan, Huiyong Wang, Yuling Zhao and Jianji Wang *

Collaborative Innovation Center of Henan Province for Green Manufacturing of Fine Chemicals, Key Laboratory of Green Chemical Media and Reactions, Ministry of Education, School of Chemistry and Chemical Engineering, Henan Normal University, Xinxiang 453007, China; lizhiyong03@126.com (Z.L.); yli@htu.cn (Y.F.); yuanxiaoqingxx@163.com (X.Y.); whyhnxx@163.com (H.W.); ylzhao@htu.cn (Y.Z.)

* Correspondence: jwang@htu.cn; Tel.: +86-373-332-5805

1. ^{1}H NMR Spectra of the Azobenzene-based Ionic Liquid Surfactants

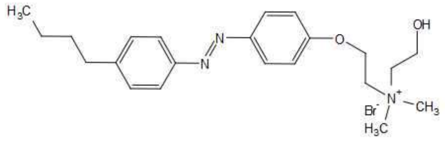

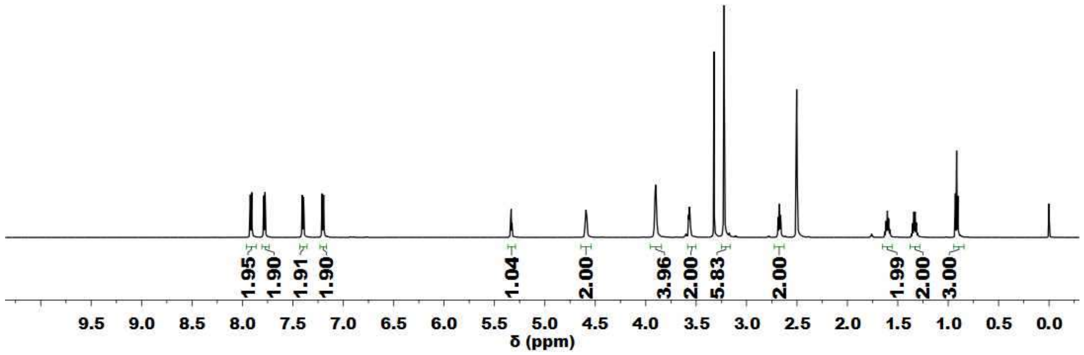

Figure S1. ^{1}H NMR spectrum of [C_4AzoC_2DMEA]Br.

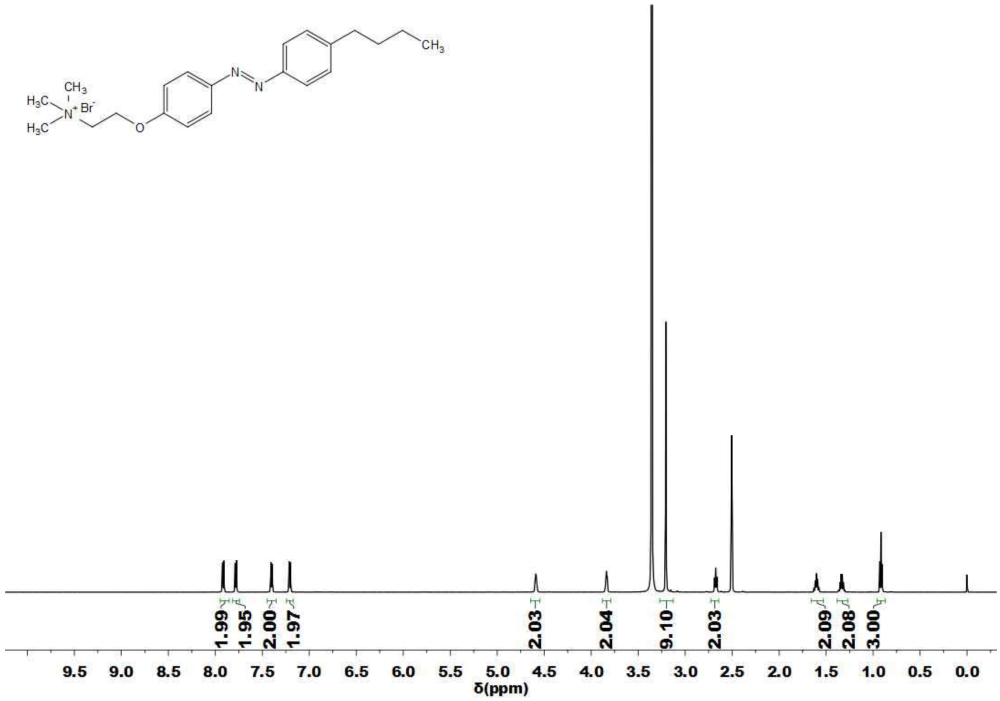

Figure S2. ¹H NMR spectrum of [C4AzoC2TMA]Br.

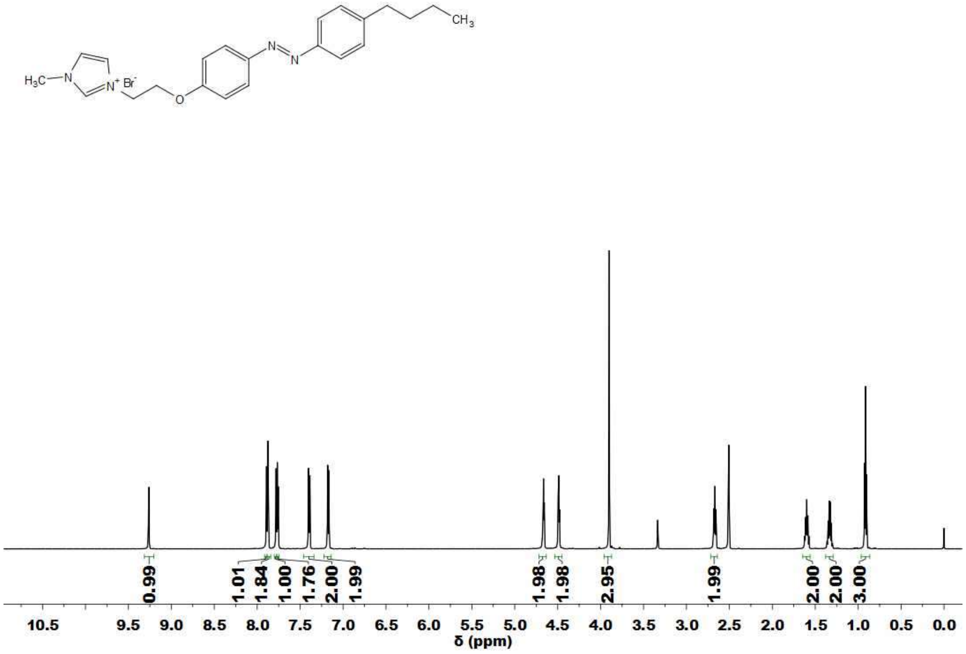

Figure S3. ¹H NMR spectrum of [C4AzoC2MIM]Br.

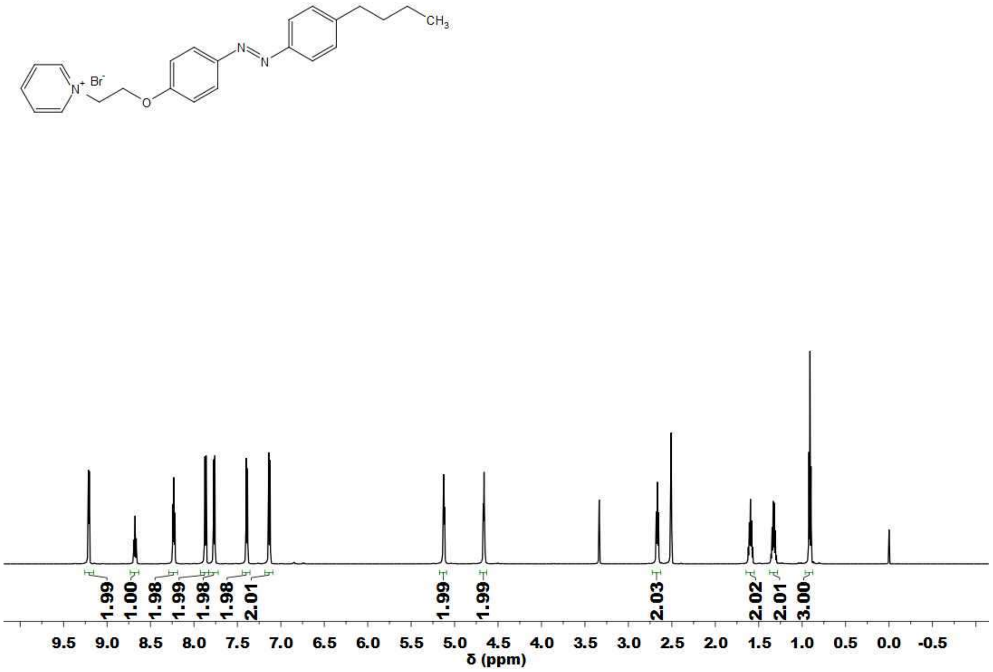

Figure S4. ^{1}H NMR spectrum of [C_4AzoC$_2$Py]Br.

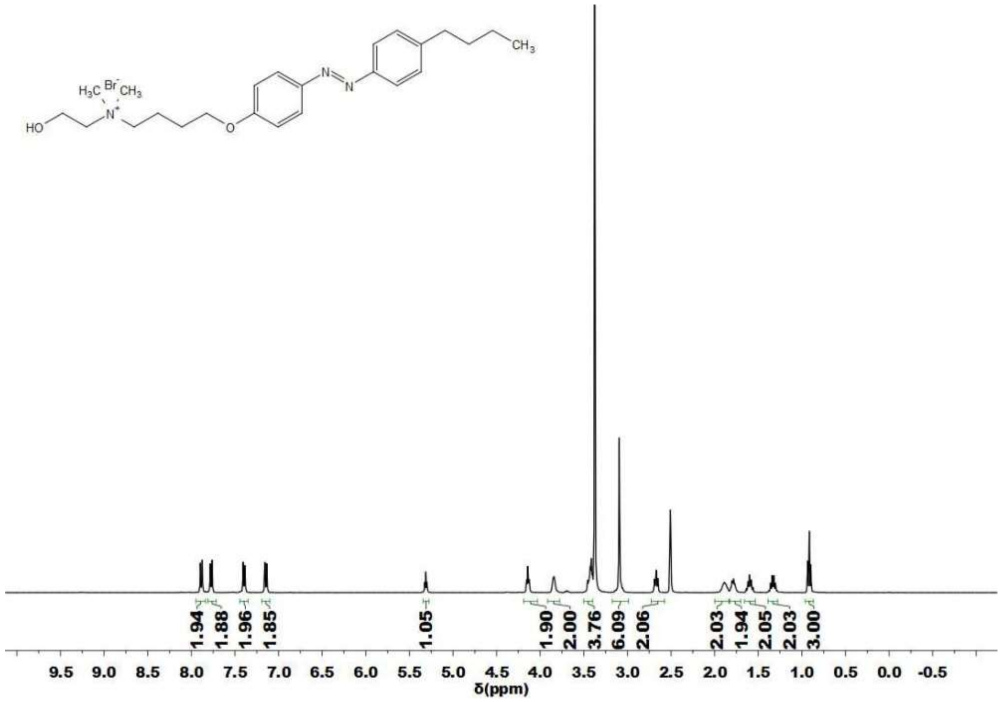

Figure S5. ^{1}H NMR spectrum of [C_4AzoC$_4$DMEA]Br.

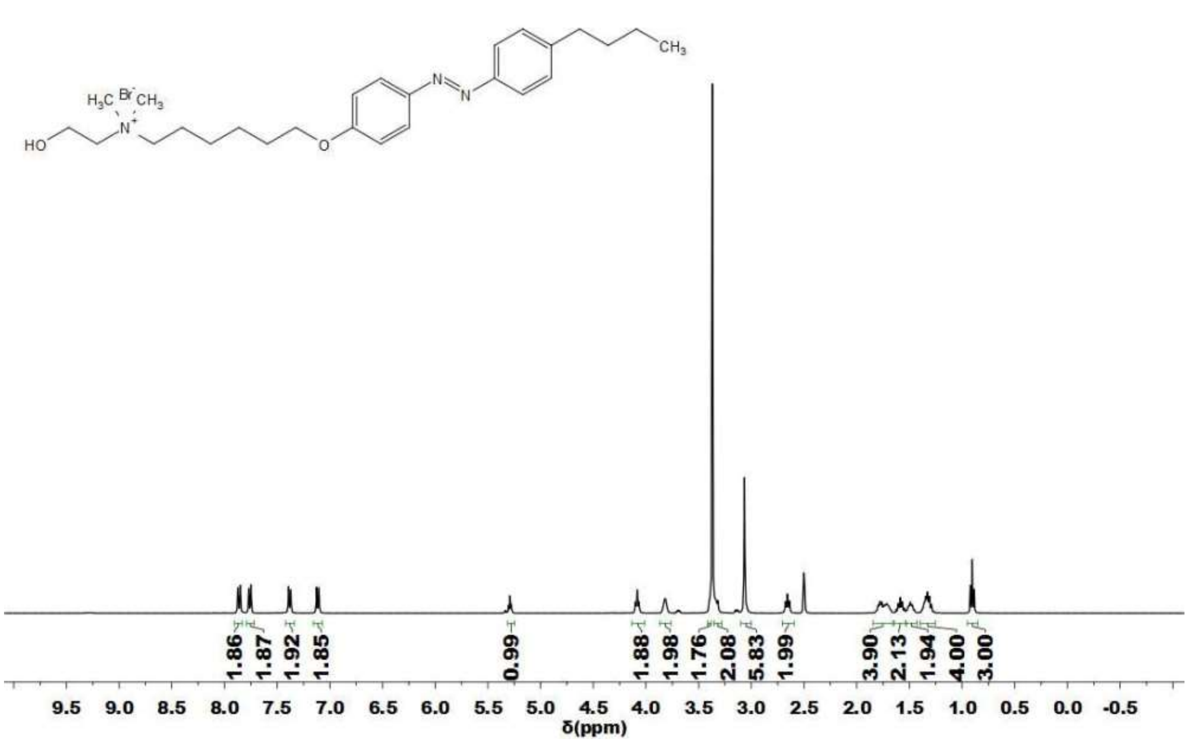

Figure S6. ^{1}H NMR spectrum of [C_4AzoC_6DMEA]Br.

2. ^{1}H NMR Data of the Azobenzene-Based Ionic Liquid Surfactants

1. **[C_4AzoC_2DMEA]Br**: ^{1}H NMR (400 MHz, DMSO-d_6, TMS): δ = 7.92 (d, 2H, Ph-H), 7.79 (d, 2H, Ph-H), 7.41 (d, 2H, Ph-H), 7.21 (d, 2H, Ph-H), 5.34 (t, 1H, -OH), 4.59 (t, 2H, -CH_2), 3.90 (t, 4H, -CH_2), 3.57 (t, 2H, -CH_2), 3.20 (s, 6H, -CH_3), 2.69 (t, 2H, -CH_2), 1.60 (m, 2H, -CH_2), 1.34 (m, 2H, -CH_2), 0.93 (t, 3H, -CH_3) ppm
2. **[C_4AzoC_2TMA]Br**: ^{1}H NMR (400 MHz, DMSO-d_6, TMS): δ = 7.91 (d, 2H, Ph-H), 7.78 (d, 2H, Ph-H), 7.41 (d, 2H, Ph-H), 7.22 (d, 2H, Ph-H), 4.59 (t, 2H, -CH_2), 3.85 (t, 2H, -CH_2), 3.20 (s, 9H, -CH_3), 2.67 (m, 2H, -CH_2), 1.60 (m, 2H, -CH_2), 1.33 (m, 2H, -CH_2), 0.91 (t, 3H, -CH_3) ppm
3. **[C_4AzoC_2MIM]Br**: ^{1}H NMR (400 MHz, DMSO-d_6, TMS): δ = 9.26 (s, 1H, imidazole ring-H), 7.89 (t, 2H, Ph-H, and 1H, imidazole ring-H), 7.78 (t, 2H, Ph-H, and 1H, imidazole ring-H), 7.40 (d, 2H, Ph-H), 7.18 (d, 2H, Ph-H), 4.67 (t, 2H, -CH_2), 4.49 (t, 2H, -CH_2), 3.90(s, 3H,-NCH_3), 2.67 (t, 2H, -CH_2), 1.61 (m, 2H, -CH_2), 1.34 (m, 2H, -CH_2), 0.93 (t, 3H, -CH_3) ppm
4. **[C_4AzoC_2Py]Br**: ^{1}H NMR (400 MHz, DMSO-d_6, TMS): δ = 9.21 (d, 2H, pyridine ring-H), 8.68 (t, 1H, pyridine ring-H), 8.23 (t, 2H, pyridine ring-H), 7.87 (d, 2H, Ph-H), 7.76 (d, 2H, Ph-H), 7.38 (d, 2H, Ph-H), 7.14 (d, 2H, Ph-H), 5.13 (t, 2H, -CH_2), 4.66 (t, 2H, -CH_2), 2.67 (t, 2H, -CH_2), 1.60 (m, 2H, -CH_2), 1.33 (m, 2H, -CH_2), 0.91 (t, 3H, -CH_3) ppm
5. **[C_4AzoC_4DMEA]Br**: ^{1}H NMR (400 MHz, DMSO-d_6, TMS): δ = 7.87 (d, 2H, Ph-H), 7.76 (d, 2H, Ph-H), 7.41 (d, 2H, Ph-H), 7.16 (d, 2H, Ph-H), 5.31 (t, 1H, -OH), 4.16 (t, 2H, -CH_2), 3.84 (t, 2H, -CH_2), 3.41 (t, 4H, -CH_2), 3.09 (s, 6H, -CH_3), 2.67 (t, 2H, -CH_2), 1.87 (m, 2H, -CH_2), 1.80 (m, 2H, -CH_2), 1.64 (m, 2H, -CH_2), 1.34 (m, 2H, -CH_2), 0.93 (t, 3H, -CH_3) ppm
6. **[C_4AzoC_6DMEA]Br**: ^{1}H NMR (400 MHz, DMSO-d_6, TMS): δ = 7.85 (d, 2H, benzene ring-H), 7.75 (d, 2H, benzene ring-H), 7.39 (d, 2H, benzene ring-H), 7.13 (d, 2H, benzene ring-H), 5.30 (t, 1H, -OH), 4.08 (t, 2H, -CH_2), 3.82 (m, 2H, -CH_2), 3.40 (m, 2H, -CH_2), 3.32 (m, 2H, -CH_2), 3.07 (s, 6H, -NCH_3), 2.66 (t, 2H, -CH_2), 1.78 (m, 4H, -CH_2), 1.59 (m, 2H, -CH_2), 1.47 (m, 2H, -CH_2), 1.31 (m, 4H, -CH_2), 0.90 (t, 3H, -CH_3) ppm

3. The UV-Vis Spectra of [C_4AzoC_2DMEA]Br in n-Octanol before and after UV Irradiation

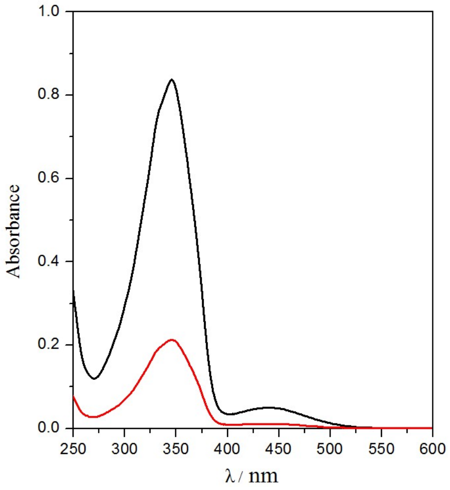

Figure S7. The UV-vis spectra of [C_4AzoC_2DMEA]Br in n-octanol: black line, before UV irradiation; red line, after UV irradiation.

4. The Reversibility of Transfer Process of [C_4AzoC_2DMEA]Br

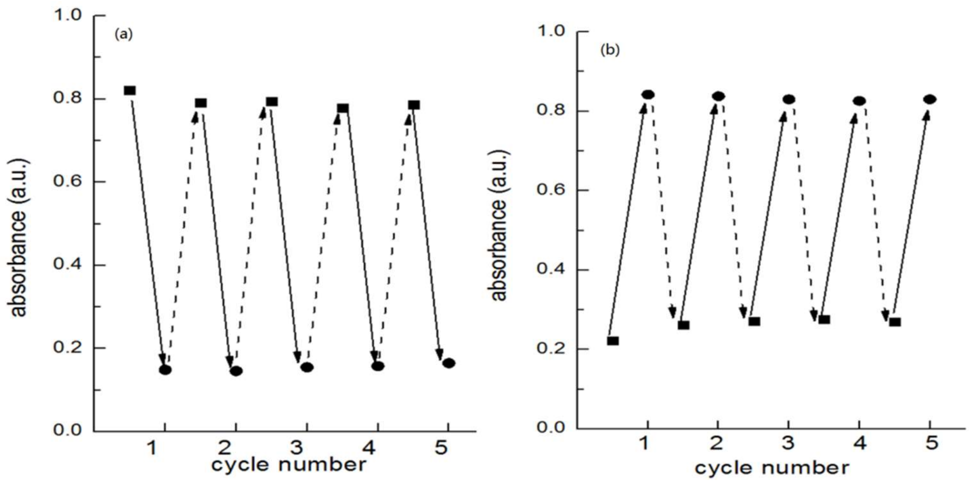

Figure S8. The reversibility of transfer process of [C_4AzoC_2DMEA]Br between organic phase and water by alternatively irradiation of UV and visible light for 5 cycles at 25.0 °C: (**a**) absorbance of [C_4AzoC_2DMEA]Br in organic phase; (**b**) absorbance of [C_4AzoC_2DMEA]Br in water; ●, after UV irradiation for 30 min; ■, after subsequent irradiation of visible light for 60 min.

5. UV-Vis Spectra for the Ionic Liquid Surfactants in n-Octanol + n-Hexane (2:1, *v*/*v*)

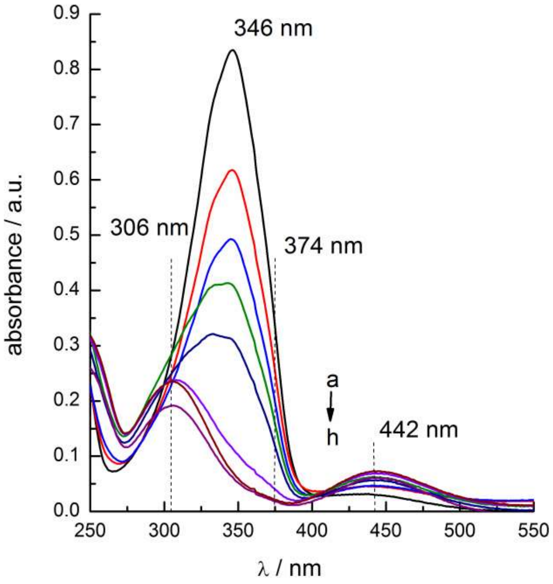

Figure S9. UV-vis spectra of 1.0 × 10^{-2} mol/kg [C_4AzoC_2MIM]Br with different UV irradiation time at 25.0 °C in n-octanol + n-hexane (2:1, *v*/*v*): a, initial state; b, 2 min; c, 4 min; d, 8 min; e, 10 min; f, 20 min; g, 30 min; h, 60 min.

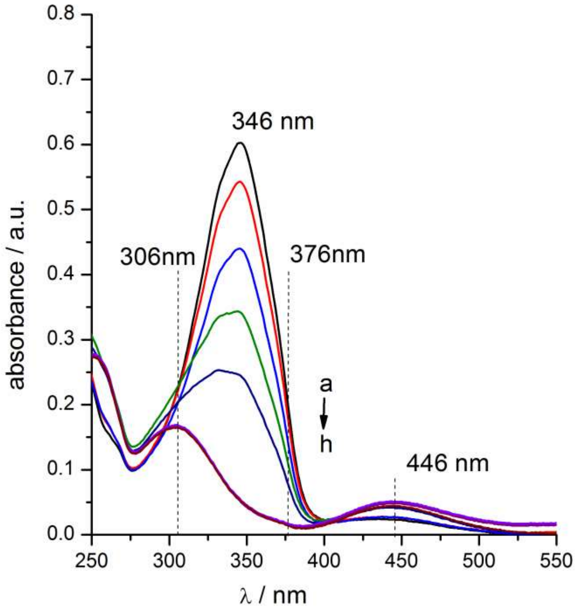

Figure S10. UV-vis spectra of 1.0 × 10^{-2} mol/kg [C_4AzoC_2Py]Br with different UV irradiation time at 25.0 °C in n-octanol + n-hexane (2:1, *v*/*v*): a, initial state; b, 2 min; c, 4 min; d, 8 min; e, 10 min; f, 20 min; g, 30 min; h, 60 min.

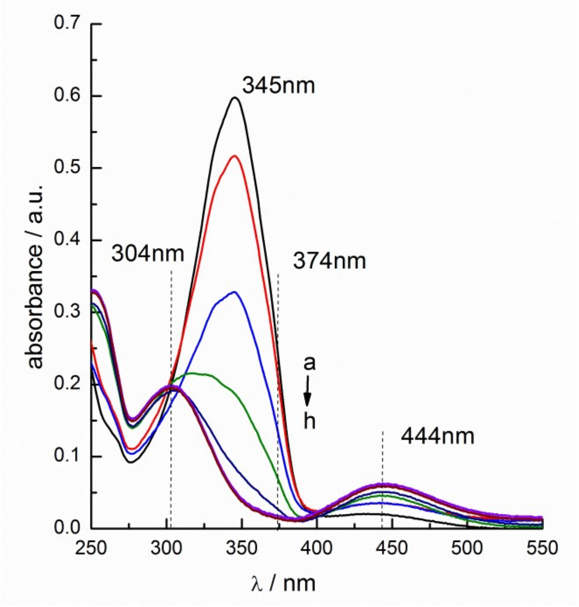

Figure S11. UV-vis spectra of 1.0 × 10^{-2} mol/kg [C_4AzoC_2TMA]Br with different UV irradiation time at 25.0 °C in n-octanol + n-hexane (2:1, *v/v*): a, initial state; b, 2 min; c, 4 min; d, 8 min; e, 10 min; f, 20 min; g, 30 min; h, 60 min.

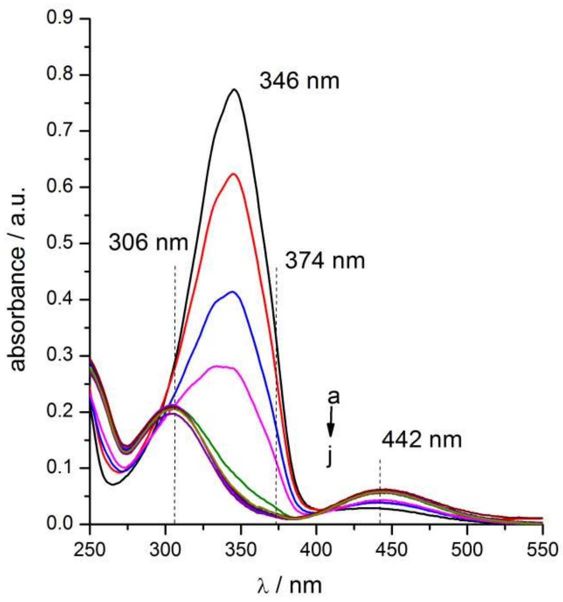

Figure S12. UV-vis spectra of 5.0 × 10^{-3} mol/kg [C_4AzoC_2DMEA]Br with different UV irradiation time at 25.0 °C in n-octanol + n-hexane (2:1, *v/v*): a, initial state; b, 1 min; c, 2 min; d, 3 min; e, 5 min; f, 7 min; g, 10 min; h, 20 min; i, 30 min; j, 60 min.

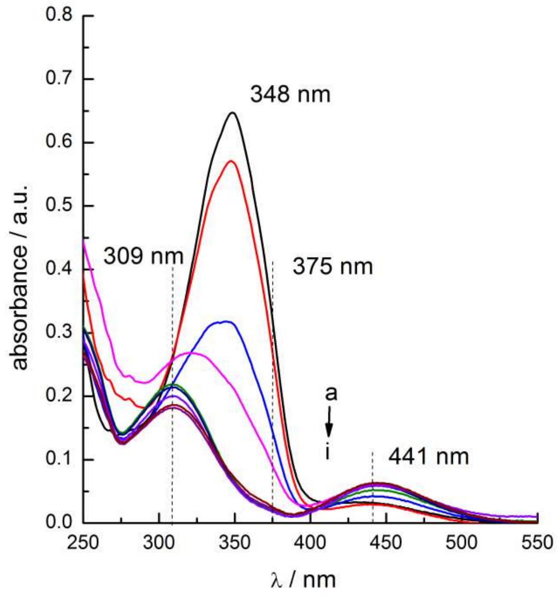

Figure S13. UV-vis spectra of 5.0 × 10^{-3} mol/kg [C_4AzoC_4DMEA]Br with different UV irradiation time at 25.0 °C in n-octanol + n-hexane (2:1, *v*/*v*): a, initial state; b, 2 min; c, 3 min; d, 5 min; e, 7 min; f, 10 min; g, 20 min; h, 30 min; i, 60 min.

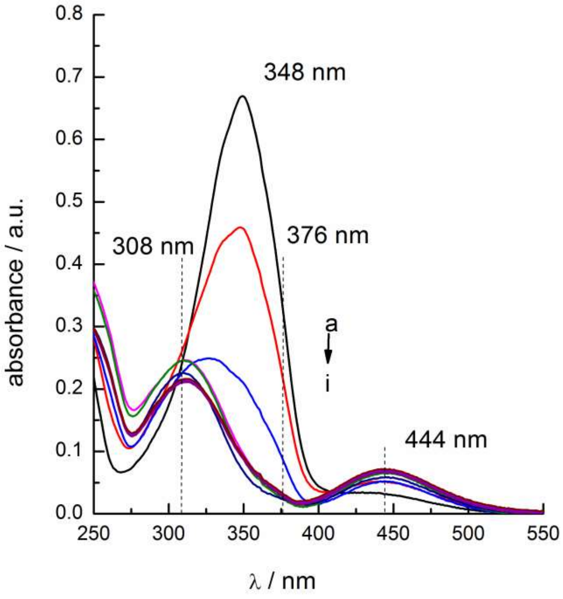

Figure S14. UV-vis spectra of 5.0 × 10^{-3} mol/kg [C_4AzoC_6DMEA]Br with different UV irradiation time at 25.0 °C in n-octanol + n-hexane (2:1, *v*/*v*): a, initial state; b, 2 min; c, 3 min; d, 5 min; e, 7 min; f, 10 min; g, 20 min; h, 30 min; i, 60 min.

6. DLS Results of the Azobenzene-Based IL Surfactants in n-Octanol/n-Hexane (2:1, *v*/*v*)

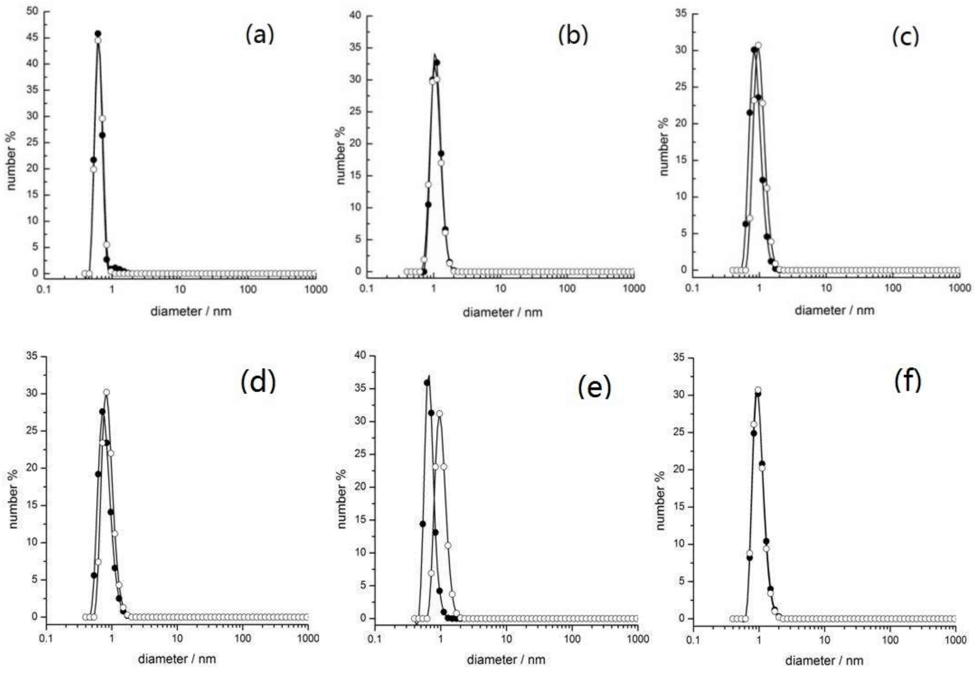

Figure S15. DLS results of 5.0 × 10^{-3} mol/kg azobenzene-based ILs in n-octanol + n-hexane (2:1, *v*/*v*) at 25.0 °C: (**a**) $[C_4AzoC_2DMEA]Br$; (**b**) $[C_4AzoC_4DMEA]Br$; (**c**) $[C_4AzoC_6DMEA]Br$; (**d**) $[C_4AzoC_2TMA]Br$; (**e**) $[C_4AzoC_2MIM]Br$; (**f**) $[C_4AzoC_2Py]Br$;●, before UV irradiation;○, after UV irradiation.

7. Critical Aggregate Concentration Determination of $[C_4AzoC_2DMEA]Br$

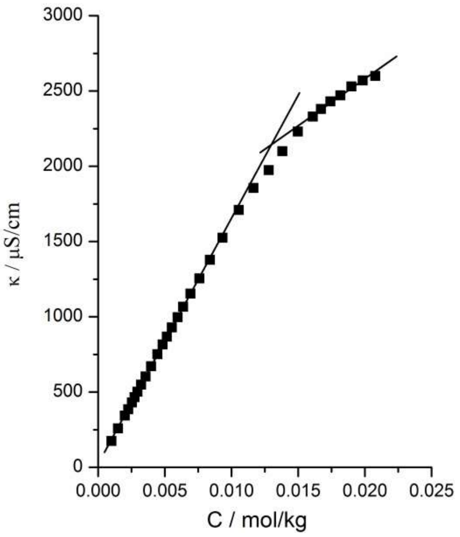

Figure S16. Conductivity of $[C_4AzoC_2DMEA]Br$ in aqueous solutions as a function of $[C_4AzoC_2DMEA]Br$ concentration at 25.0 °C after UV irradiation.

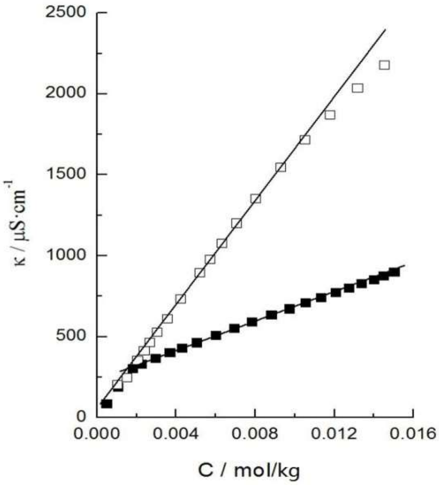

Figure S17. Conductivity of [C_4AzoC_2Py]Br in aqueous solutions as a function of [C_4AzoC_2Py]Br concentration at 25.0 °C: ■, before UV irradiation; □, after UV irradiation.

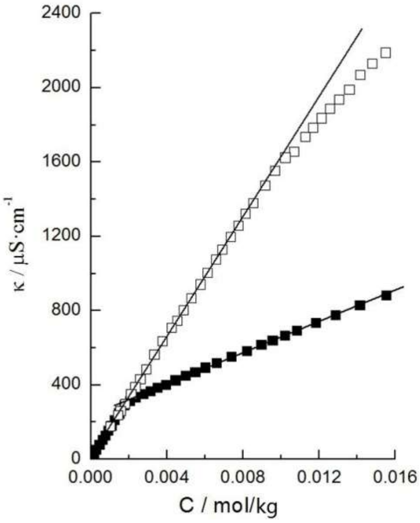

Figure S18. Conductivity of [C_4AzoC_2MIM]Br in aqueous solutions as a function of [C_4AzoC_2MIM]Br concentration at 25.0 °C: ■, before UV irradiation; □, after UV irradiation.

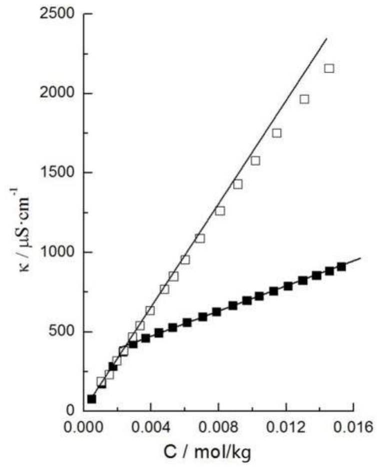

Figure S19. Conductivity of [C_4AzoC_2TMA]Br in aqueous solutions as a function of [C_4AzoC_2TMA]Br concentration at 25.0 °C: ■, before UV irradiation; □, after UV irradiation.

8. Solubility of the Azobenzene-Based Compounds in Water

Table 1. Solubility of the azobenzene-based compounds in water at 25.0 °C.

Compound	Solubility/mol/L
[C_4AzoC_2MIM]Br	0.98 [a]
[C_4AzoC_2Py]Br	8.66×10^{-2}
[C_4AzoC_2TMA]Br	0.86 [a]
[C_4AzoC_2DMEA]Br	0.94 [a]
[C_4AzoC_4DMEA]Br	0.93 [a]
[C_4AzoC_6DMEA]Br	1.05 [a]

[a] At this concentration, the gel formed in the system and the viscosity increased significantly. In this case, it is hard to continue the dissolution.

9. The Melting Point of the Azobenzene-Based Compounds

Table 2. The melting point of azobenzene-based compounds.

Compound	Melting Point/°C
[C_4AzoC_2MIM]Br	147
[C_4AzoC_2Py]Br	167
[C_4AzoC_2TMA]Br	147
[C_4AzoC_2DMEA]Br	154
[C_4AzoC_4DMEA]Br	82
[C_4AzoC_6DMEA]Br	93

© 2019 by the authors. Submitted for possible open access publication under the terms and conditions of the Creative Commons Attribution (CC BY) license (http://creativecommons.org/licenses/by/4.0/).
